# Supplementary figures and images for: The chemical composition of a new “mica sandwich” foraminiferal species from the East Coast of Korea: Capsammina crassa sp. nov
Source: PeerJ. 2019 Mar 21;7:e6642. doi: 10.7717/peerj.6642 (PMC6431544; doi:10.7717/peerj.6642)

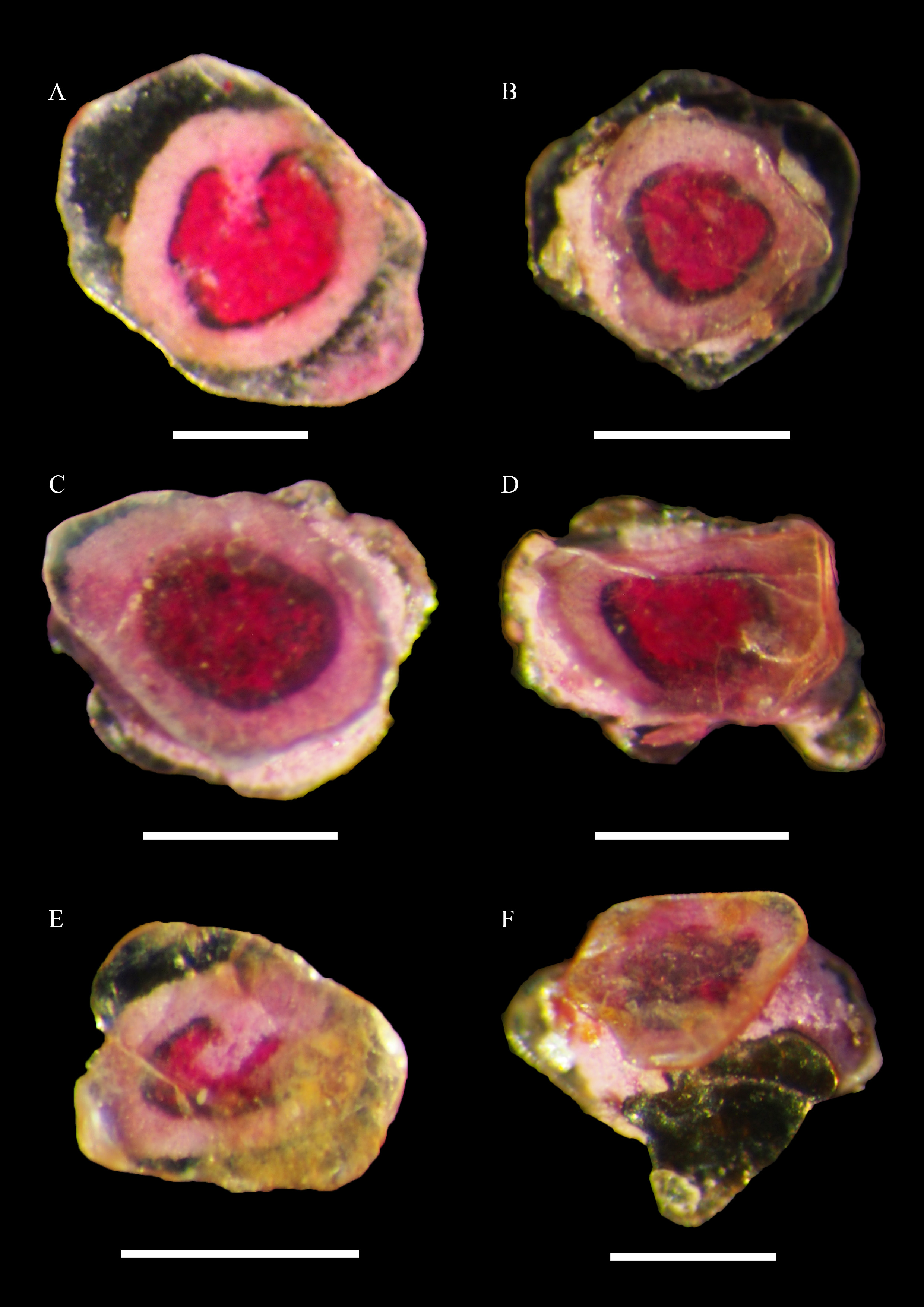

Supplement: Supplemental Information 4 — Light micrographs of Rose Bengal stained specimens. The holotype is shown in C; the remaining specimens are paratypes. Scale bars = 500 μm. [file peerj-07-6642-s004.jpg]

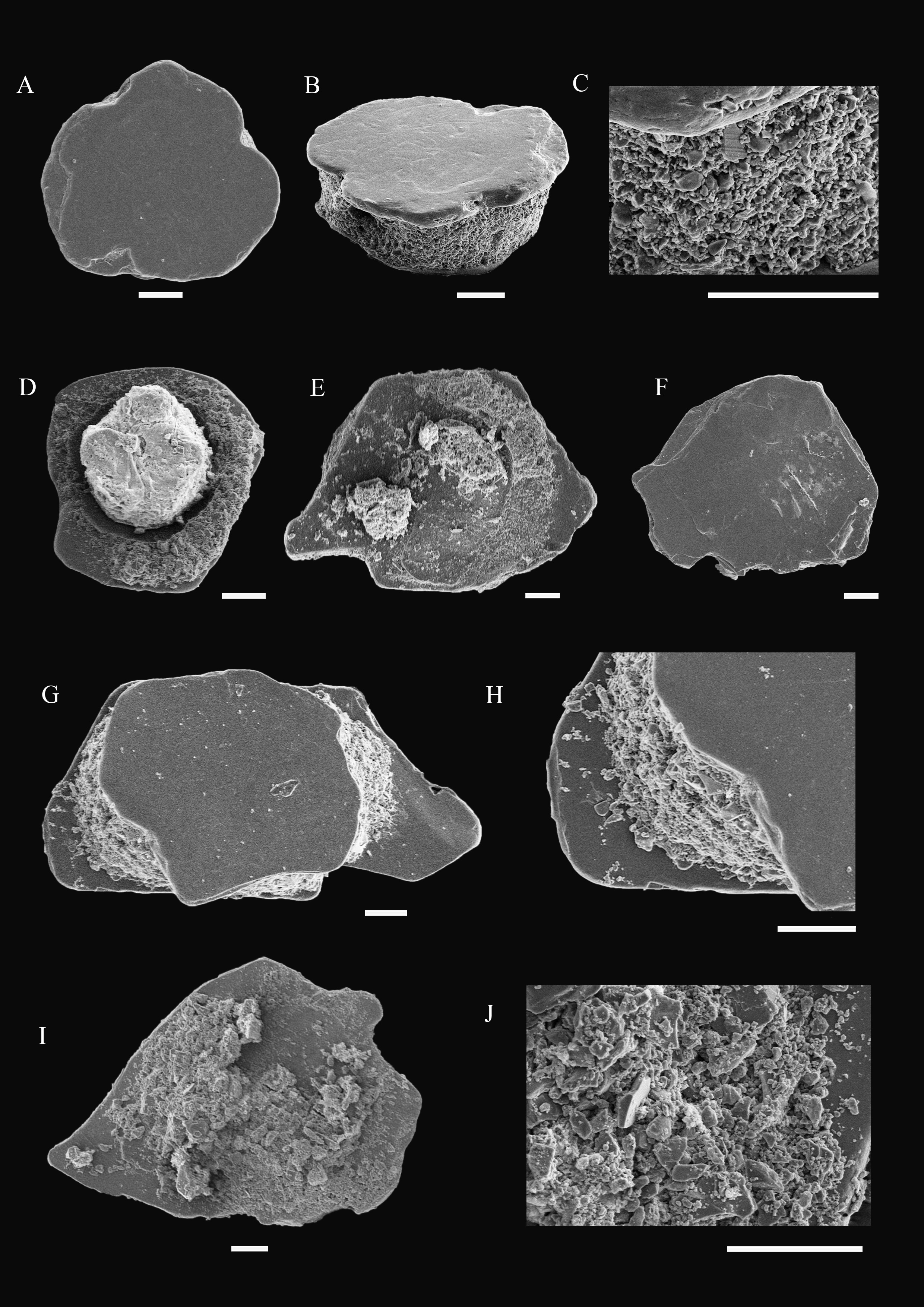

Supplement: Supplemental Information 5 — A. View of the flat external surface of mica plate. B. Side view; C. Close-up of agglutinated mineral grains between mica plates. D. Test with one mica plate detached. Central lighter part is the cell body. E. Detached mica plate with the remains of the ring of finely agglutinated grains adhering to the surface. F. Outer surface of mica plate. G. Intact test. H. Detail showing finely agglutinated wall between two mica plates. I. Underside of detached mica plate. J. Detail of agglutinated grains adhering to the underside of the plate. Scale bars = 100 μm. [file peerj-07-6642-s005.jpg]
